# Supplementary material for: MetaRibo-Seq measures translation in microbiomes
Source: Nat Commun. 2020 Jun 29;11:3268. doi: 10.1038/s41467-020-17081-z (PMC7324362; doi:10.1038/s41467-020-17081-z)
Supplement: Supplementary file 10 — Supplementary Data 7 [file 41467_2020_17081_MOESM10_ESM.zip › File2/Confidence_VeryHigh_Taxonomy/349020_out.krona.html]

Javascript must be enabled to view this page.

members
magnitude
magnitudeUnassigned
count
unassigned
taxon
rank

349020\_out

14

superkingdom
1
2759

kingdom
1
4751

451864
1
subkingdom

1
5204
phylum

subphylum
1
5302

class
155619
1

1
452335
subclass

order
1
157608

68784
1
family

1
68785
genus

species
68786
1

SRS143523\_contig\_number\_contig-100\_1933.145362

13
2
superkingdom

13
1239
phylum

class
5
186801

order
5
186802

family
4
186803

841
1
genus

species
1

SRS018313\_contig\_number\_1395
2292368

genus
1407607
1


SRS144135\_contig\_number\_contig-100\_1063.192018
1
1150298
species

species

SRS052697\_contig\_number\_contig-100\_1750.218355SRS062654\_contig\_number\_9834
2
1898203

541000
1
family

1263
1
genus

species
1
46228

SRS052697\_contig\_number\_25627

8
1263012

SRS015694\_contig\_number\_9440SRS016203\_contig\_number\_contig-100\_547.74969SRS024435\_contig\_number\_contig-100\_7.247898SRS098644\_contig\_number\_50308SRS142503\_contig\_number\_49598SRS143876\_contig\_number\_35381SRS147022\_contig\_number\_14746SRS893358\_contig\_number\_13861
species
